# HIV pre-exposure prophylaxis during the SARS-CoV-2 pandemic: Results from a prospective observational study in Germany

**DOI:** 10.3389/fpubh.2022.930208

**Published:** 2022-08-24

**Authors:** M. Uhrmacher, A. Skaletz-Rorowski, S. Nambiar, A. J. Schmidt, P. Ahaus, K. Serova, I. Mordhorst, A. Kayser, J. Wach, C. Tiemann, D. Münstermann, N. H. Brockmeyer, A. Potthoff

**Affiliations:** ^1^WIR – Walk in Ruhr, Center for Sexual Health and Medicine, Bochum, Germany; ^2^Interdisciplinary Immunological Outpatient Clinic, Center for Sexual Health and Medicine, Department of Dermatology, Venereology and Allergology, Ruhr Universität Bochum, Bochum, Germany; ^3^Sigma Research, Department of Public Health, Environments and Society, London School of Hygiene and Tropical Medicine, London, United Kingdom; ^4^Division of Infectious Diseases, Cantonal Hospital St. Gallen, St. Gallen, Switzerland; ^5^Institute of Educational Research, Ruhr Universität Bochum, Bochum, Germany; ^6^Aidshilfe Bochum e.V. (Aids-Service Organization Bochum), Bochum, Germany; ^7^Local Health Department Bochum, Bochum, Germany; ^8^Laboratory Krone, Bad Salzuflen, Germany

**Keywords:** HIV, pre-exposure prophylaxis, MSM, SARS-CoV-2, pandemic (COVID-19), sexual behavior, Chemsex drugs, sexual transmission infection

## Abstract

**Aims:**

Since 2017, HIV pre-exposure prophylaxis (PrEP) care has been provided through an intersectoral collaboration at WIR (Walk-in-Ruhr, Center for Sexual Health and Medicine, Bochum, Germany). The aim of this study was to establish possible impact of COVID-restrictions on the sexual behavior of PrEP users in North Rhine-Westphalia.

**Methods:**

The current PrEP study collected data of individuals using PrEP, their sexual behavior and sexually transmitted infections (STIs) before (each quarter of year 2018) and during the COVID-19 pandemic (each quarter of year 2020).

**Results:**

During the first lockdown in Germany from mid-March until May 2020, PrEP-care appointments at WIR were postponed or canceled. Almost a third of PrEP users had discontinued their PrEP intake in the 2^nd^ quarter of 2020 due to alteration of their sexual behavior. The number of sexual partners decreased from a median of 14 partners in the previous 6 months in 1^st^ quarter of 2020, to 7 partners in 4^th^ quarter of 2020. Despite such a significant reduction in partner number during the pandemic in comparison to the pre-pandemic period, a steady rate of STIs was observed among PrEP users in 2020.

**Conclusion:**

The SARS-CoV-2-pandemic has impacted PrEP-using MSM in North Rhine-Westphalia with respect to their PrEP intake regimen and sexual behavior in 2020. Our study revealed a steady rate of STI among PrEP users even during the pandemic, thus highlighting the importance of ensuring appropriate HIV/STI prevention services in times of crisis.

## Introduction

In 2019, 3,111 new HIV infections were diagnosed in Germany, 8% more than in 2018 (1). For the last 10 years, more than 2,500 new HIV diagnoses occurred yearly in Germany and the number of people living with HIV has been consistently increasing. The majority of transmissions of HIV occurred among men who have sex with men (MSM) (2). Germany, together with other countries, is striving to end the epidemic of AIDS by 2030, a goal that has been set by the international community in the Agenda for Sustainable Development (3). Therefore, the prevention of HIV continues to be a major public health challenge. In addition to behavioral interventions like promoting condom use among sexually active populations, a new form of biomedical HIV prevention, the HIV pre-exposure prophylaxis (PrEP) is also employed among populations at high risk for HIV. As evidenced from clinical trials and observational studies, PrEP has been proven to be highly effective among people at substantial HIV risk (4–6).

In Germany, PrEP became available in 2016 with the antiretroviral drugs tenofovir disoproxil fumarate (TDF) and emtricitabine (FTC). German/Austrian PrEP guidelines define people with substantial HIV risk as MSM or transgender women engaging in condomless anal intercourse within the previous 6 months, those with sexually transmitted infections (STIs) within the previous 12 months, and/or having HIV-positive sex partners with insufficient antiretroviral control / suppression (7). In the initial years of PrEP availability in Germany, the official costs for users were over 500€ for 30 pills, but since the arrival of a generic in August 2017, PrEP became affordable at 50€ per month. Since September 2019, PrEP costs are covered by German statutory health insurance. PrEP prescription mandates a prior professional counseling on HIV preventive measures, surveillance of other STIs and kidney parameters and excluding the possibility of a pre-existing HIV infection (6).

The *Walk In Ruhr* (WIR) is a center for sexual health and medicine in Bochum, offering cross-sectoral PrEP care: clinicals of the department of dermatology, venereology, and allergology at *Ruhr University Bochum, Aidshilfe Bochum* (an Aids service NGO) and the city's public health department. By combining prevention services of those institutions and offering discreet and early appointments, the WIR follows its aim of reducing threshold of STI prevention. During the coronavirus disease 2019 (COVID-19) pandemic, the WIR, as a health care center for sexual medicine, had to adjust the modality of health work to maintain low-threshold accessibility.

First cases of COVID-19 in Germany were reported on 27 January 2020 (8). From 13 March, a lockdown was introduced including closures of public institutions like universities, schools and kindergarten. Social contacts were additionally reduced by the imposition of mandatory contact restrictions and curfews (8). As the SARS-CoV-2 infection rate decreased, a relaxation of restrictions took place from early May and during the summer. By September 2020, higher infection numbers indicating a second wave of the pandemic led to even stricter physical distancing rules starting on November 2^nd^. Educational institutions remained open until stronger lockdown measures were imposed on December 16^th^. In Germany, the vaccination campaign was initiated on December 26^th^. Until the end of 1^st^ quarter in 2021, 11% of the population had received the 1^st^ and 5% the 2^nd^ dose of the vaccine (9) while infection numbers continued to increase and contact restrictions were still ongoing. During the first lockdown in the 2^nd^ quarter of 2020, patient contact in the WIR was reduced to a minimum. PrEP follow-up appointments were canceled or postponed, and only patients with acute symptoms or medical issues were admitted to our clinic. PrEP users requiring a new prescription were provided with an appointment by phone to discuss their PrEP regimen, side effects and possible STI symptoms with a physician. The prescription was sent by mail. With the relaxation of general contact restrictions in May, the WIR resumed to offer all health care and prevention facilities, among them, PrEP initiation and follow-up appointments.

In this paper, we present longitudinal data from a monocentric survey of PrEP users during the COVID-19 pandemic in Germany in 2020, in comparison with pre-pandemic data from 2018. The main objectives of our study are to investigate/examine:

- the impact of COVID-19 pandemic on PrEP intake regimen among a cohort of PrEP-using MSM.
- the impact of COVID-19 pandemic on the sexual behavior (number of partners, STIs, condom use, substance use) of PrEP-using MSM in comparison to the pre-pandemic period (2018).
- whether COVID-driven reduction of social contacts in general also lead to a concomitant reduction in sexual contacts among PrEP-using MSM.
- any association between oral use of PrEP and decreased infectability to COVID-19.

Availability of such information on changes to access and utilization of our sexual health care center (Ruhr area, Germany) will enable the global scientific community to contextualize it with similar reports from other regions, thus enabling a more global overview of challenges in HIV and STI prevention care during times of crisis.

## Materials and methods

### Study design and population

Data were obtained from an open, monocentric, prospective, observational study of individuals receiving HIV PrEP at WIR since September 2017 and by analyzing sociodemographic factors of PrEP users, the numbers of sexual partners, drug use and laboratory-confirmed STIs (10). Included in our research were 138 PrEP users that began using PrEP after German statutory health insurance started to cover the costs of PrEP in September 2019 until the first lockdown in March 2020. For every PrEP user the observation period was 13 months (between September 2019 and March 2021). Inclusion criteria were being MSM and a sufficient knowledge of the German language.

Participants who did not attend a follow-up after the first lockdown were contacted by a recall system and invited to an alternative appointment including STI-testing. If the proposal of further follow-ups was declined, the current PrEP regimen was assessed by phone, and the reasons for discontinuation of follow-up appointments at WIR and pausing PrEP were discussed.

For comparison to a pre-pandemic year, data was obtained from a previous PrEP cohort at WIR between October 2017 to December 2018 (10) who were similarly observed for a period of 13 months.

### Data collection

Data was collected at six time-points: the date of first PrEP prescription and five follow-up visits (appointments in months - 1, 4, 7, 10 and 13 of PrEP use). All PrEP users were provided with a 34-item questionnaire at time of initiation, and a subsequent 15-item questionnaire during follow-up. With every follow-up, clinical data like renal parameters, results of STI-testing, prescription for STI treatment, and new vaccinations against hepatitis A and B were evaluated.

In addition to the existent questionnaire, a new questionnaire was introduced in May 2020 for (1) evaluating the risk for a SARS-CoV-2-infection (2) pandemic-related changes in sexual behavior and (3) pandemic associated changes in the number of both sexual and non-sexual contacts. Adherence and the current PrEP regimen were documented for each participant at each follow-up visit.

### Diagnosis of HIV and other STIs

Incidences of HIV and other STIs were recorded at the time of PrEP initiation and with follow-up appointments (in Month 4, 7, 10 and 13) according to German clinical guidelines: HIV RNA (only at baseline), 4^th^ generation HIV antibody test, viral p24-antigen, antibodies against the hepatitis A virus (HAV-AB), the hepatitis B surface antigen (HBs-AB), and the hepatitis B core (HBc-AB), TPPA [Treponema pallidum particle agglutination assay], VDRL (Venereal Disease Research Laboratory). Infections with *Neisseria gonorrhoeae* (*NG), Chlamydia trachomatis* (*CT)* and *Mycoplasma genitalium* (*MG)* were tested by self-collected oral and rectal swabs and first urine for Nucleic Acid Amplification Test (Cobas CT/NG, Roche Diagnostics, Mannheim, Germany; Cobas TV/MG, TIB Molbiol, Roche Diagnostics, Mannheim, Germany). All participants with incident infections of CT, NG, MG or TV received a test of cure (TOC) six weeks after diagnosis. In addition, at each visit, diagnosed STIs outside this study were recorded. A vaccination against HAV or HBV was offered to participants with no or insufficient antibodies. According to German guidelines, sufficient immune response was defined as anti-HAV-IgG >20 U/l, and anti-HBs >100 U/l, respectively.

### Diagnosis of antibodies against SARS-CoV-2

From November 2020 to March 2021, a test for antibodies against SARS-CoV-2 was offered to the study participants to establish SARS-CoV-2 serostatus. Serum samples were analyzed with ELISA for SARS-CoV-2 IgG using EUROIMMUN (#EI 2606-9601G). The CE-marked test has a reported sensitivity of 94.4% (>10 days after onset of symptoms or positive direct detection) and a specificity of 99.6% (11).

### Ethical considerations

Ethics approval was obtained from the Ethics Committee of the Medical Faculty at Ruhr-Universität Bochum (Request-number 17-6208-BR) and all participants provided informed consent.

### Statistical analysis

Descriptive statistics were used to calculate the proportions of PrEP visits, PrEP regimen, number of sexual and non-sexual partners, and diagnosed STIs for each quarter of 2020. For the purpose of comparison, data was compiled in absolute counts and percentages. All statistical analyses were performed with Microsoft Office Excel and R version 4.0.3 (2020-10-10) “Bunny-Wunnies Freak Out,” © 2020 The R Foundation for Statistical Computing.

Descriptive statistics were used to describe the characteristics of the sample. Differences regarding the PrEP regimen between the two cohorts in 2018 and 2020 were tested by Fisher's exact test and by chi-square test for categorical variables. Differences in means of partner numbers and proportions of positive STI tests between quarters were analyzed using Wilcoxon signed-rank test for paired measurements and Mann-Whitney *U* for independent groups. To adjust for the problem of alpha-error-cumulation, Holm-Bonferroni method was used with *p*-values of consecutive statistical tests. To elaborate correlations between a job-related contact reduction and a decrease of sexual partners, contingency tables and chi-square tests were performed. *P*-values lower than 0.05 were considered to be statistically significant, based on two-sided tests.

## Results

### Sociodemographic data of PrEP users before and in time of SARS-CoV-2 pandemic

Overall, a total of 144 individuals visited WIR to start PrEP between September 2019 and March 2020. Five of them did not start PrEP because of a self-assessed low risk for HIV infection. One female PrEP user was excluded from the study, to achieve an exclusively MSM PrEP-using study cohort of *n* = 138.

Before German statutory health insurance started to reimburse the costs of PrEP in September 2019, an average of 30–40 PrEP initiations occurred at the WIR every quarter. In contrast, during the 4^th^ quarter of 2019 and the 1^st^ quarter of 2020, 66 and 58 new PrEP users were registered. After the interruption of PrEP initiations (during the 2^nd^ month of the 2^nd^ quarter) due to the first lockdown in 2020 at WIR, 26 new PrEP users were registered at WIR in May and June 2020 until the end of that quarter. From July to September (3^rd^ quarter), 40 PrEP initiations were registered, and from October until the end of 2020 (4^th^ quarter) 26 new PrEP initiations were registered respectively. Overall, 150 new PrEP users were documented at WIR in 2020. In comparison, during 2018, when PrEP costs had not been incurred by statutory health insurance only 125 PrEP initiations had been documented.

After the first lockdown, 37 (26.8%) participants did not report for an in-person appointment at WIR during summer 2020. By a recall system that had been implemented in October 2020, they were notified and invited to another appointment. Until 1^st^ quarter of 2021, 35 of them were contacted successfully and 14 new PrEP appointments could be scheduled.

Sociodemographic data of the PrEP users are shown in Table 1.

**Table 1:** Sociodemographic data and general characteristics of PrEP users in 2020.

		***N* (%)**
Sex	Male	138 (100)
	Female	0 (0)
Mean age *(SD)*		33.9 (± 10.1)
Gender of sexual partners	Male	138 (100)
	Female	7 (5.0)
	Transgender	5 (3.6)
School years	12 or more	100 (72.5)
	10 or 11	37 (26.8)
	Up to 9	1 (0.7)
Profession	Employed (or self-employed)	89 (64.5)
	In formation (school/university/training program)	35 (25.4)
	Retired	5 (3.6)
	Unemployed	6 (4.3)
	Other work situation	3 (2.2)
Income	Own income	117 (84.8)
	Financial support during formation by parents/ state /credit	14 (10.1)
	Pension	3 (2.2)
	Unemployment benefit	3 (2.2)
	State income support	1 (0.7)
Health insurance	Statutory	132 (95.7)
	Private	6 (4.3)
Country of birth	Germany	119 (86.2)
	Other EU country	9 (6.5)
	Other non-EU country	10 (7.2)
Transactional sex (before PrEP initiation)	Has been paid	4 (2.9)
	Did pay	3 (2.2)
PrEP regimen	Daily	128 (92.8)
	Intermittent	9 (6.3)
	n/a	1 (0.7)
Previous HIV chemoprophylaxis	PEP	11 (7.7)
	PrEP	15 (10.4)
Completed vaccinations *n (%)*	Hepatitis A (2 dosages)	82 (56.9)
	Hepatitis B (3 dosages)	81 (56.3)
Previous STIs	Yes	86 (62.3)

*SD, standard deviation; STIs, sexually transmitted infections; PEP, post-exposure prophylaxis; PrEP, pre-exposure prophylaxis; n/a, not applicable*.

### PrEP regimen

**Figure 1:**
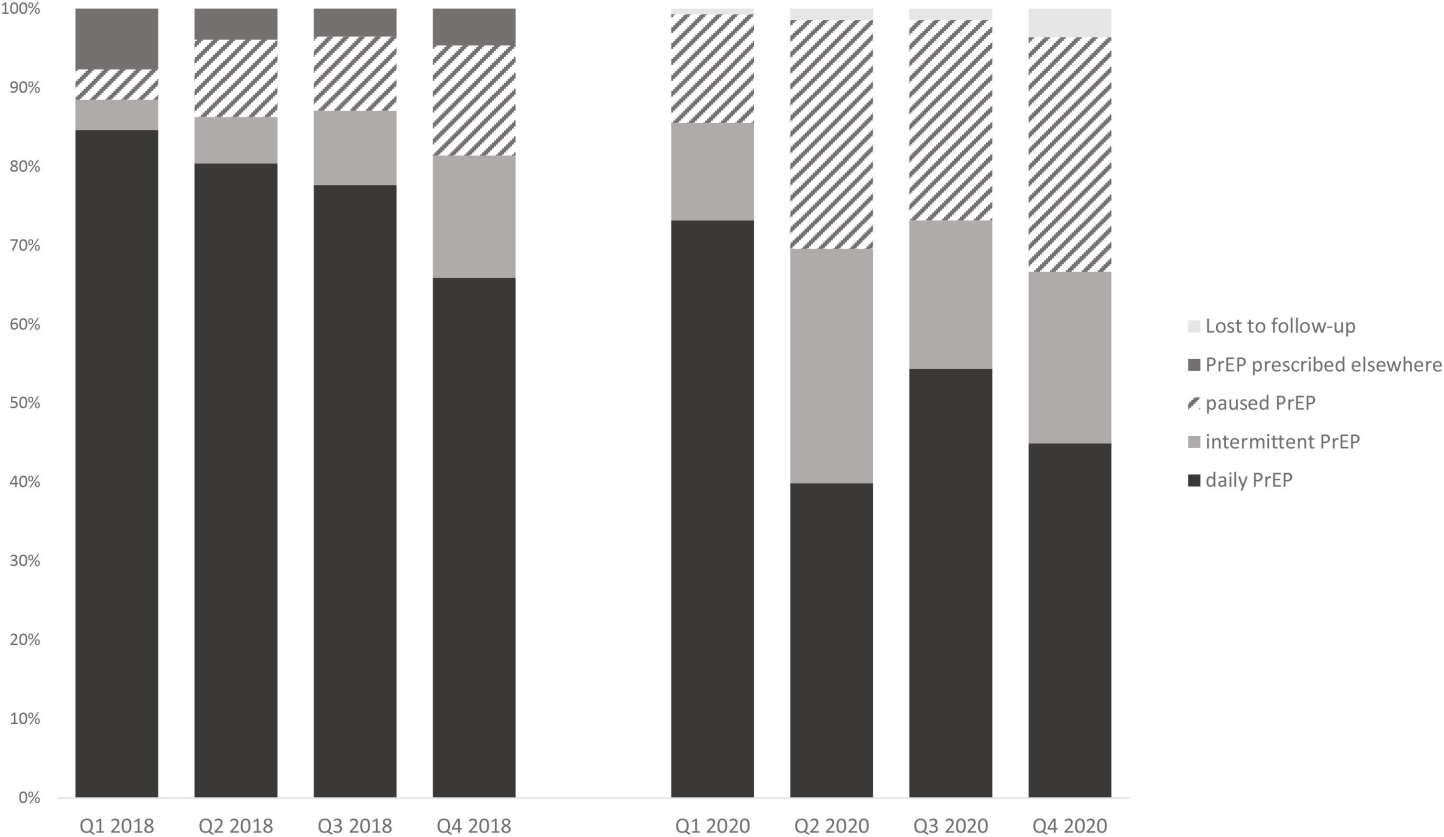
PrEP intake regimen in the four quarters of 2018 vs. 2020.

As shown in Figure 1, of the 138 subjects who initiated PrEP prior to the COVID-19 pandemic, 40 (29.0%) had already terminated or paused PrEP medication in the 2^nd^ quarter of 2020. In the 3^rd^ quarter, fewer users paused PrEP [35 (25.4%)] until further users stopped again during the second lockdown in the 4^th^ quarter [41 (29.7%)]. In comparison, individuals who initiated PrEP in 2017/18 demonstrated an increasing trend in PrEP discontinuation / pause. Eighteen users (13.9%) had stopped PrEP by the end of 2018. The comparison of the PrEP intake in 2018 vs. 2020 showed significant differences during the months of lockdown in 2^nd^ quarter of 2020 *[X*^2^*(3)* = *27.959, p* < *0.001*, ϕ = 0.45*]*, with a higher proportion of both PrEP discontinuation and pause toward the end of 2020 *[X*^2^*(3)* = *14.12, p* = *0.003*, ϕ = *0,31]*.

A similar trend was also observed in the switch from daily to intermittent PrEP: Compared to 2018, more participants reported intermittent PrEP in 2020, especially during the months of lockdown [2^nd^ quarter, 51 (29.7%); 4^th^ quarter, 30 (21.7%)]. In 2018, more users generally used daily PrEP, and by 4^th^ quarter 2018 the number of intermittent PrEP users had increased only slightly to 15.5%.

### Numbers of sexual partners in the previous 6 months

**Figure 2:**
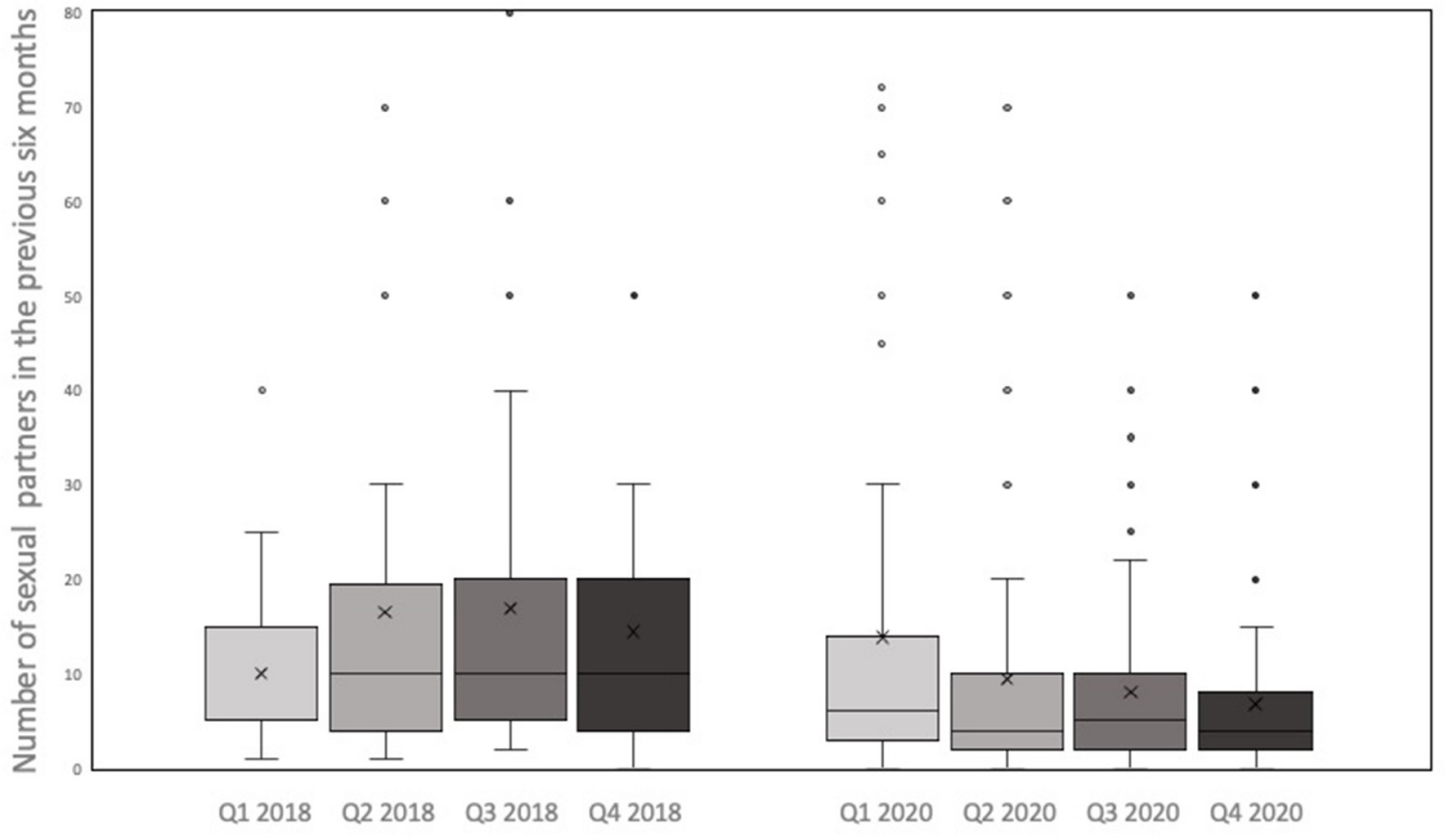
Boxplots: number of sexual partners in the four quarters of 2018 vs. 2020.

**Figure 3:**
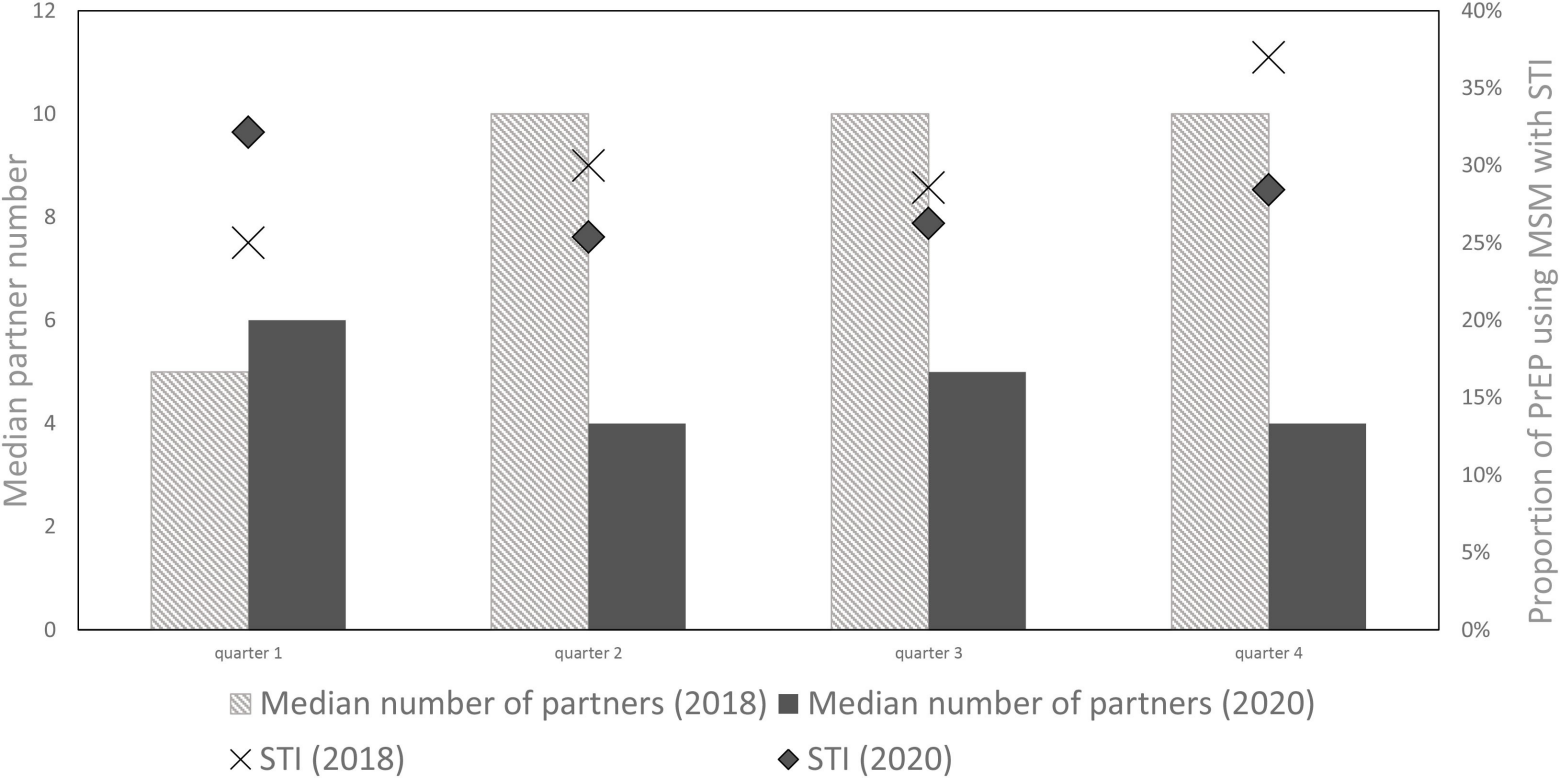
Median partner number and proportion of PrEP using MSM with positive STI tests (%) in 2018 and 2020.

At each PrEP appointment, participants were asked about the numbers of sexual partners in the previous 6 months (see Figures 2, 3).

In 2020, the minimum number of sexual partners was 0 for all quarters, while the maximum number decreased from 200 partners in 1^st^ quarter to 70 in the 2^nd^ and 50 in the 3^rd^ and 4^th^ quarter, respectively. The mean number of reported sexual partners significantly decreased from 14 in the 1st quarter to 9 in the 2nd quarter of 2020 *(z* = −*4.392, p* = < *0.001, r* = *0.366)*. In the subsequent quarters the number of sexual partners did not increase again. In contrast, in 2018, no significant change in the mean or median number of sexual partners between consecutive quarters could be observed *(p* = *0.372; p* = *0.51; p* = *0.668)*. On comparing the number of sexual partners of PrEP users in the 2^nd^ quarter of 2018 vs. 2020, participants reported significantly fewer partners during the lockdown of 2020 *(z* = −*3,363, p* < *0.001, r* = *0.280)*.

### Reduction of non-sexual contacts

To understand changes in number of sexual partners in the context of adherence to social distancing measures, PrEP users were further surveyed using an amended questionnaire after the first lockdown. This questionnaire interrogated them on both the number of pandemic-associated professional/ private non-sexual physical contacts as well as pandemic-associated sexual contacts. Among them, 34 (32.4%) reported reduced number of non-sexual physical contacts, 3 (2.9%) had more non-sexual physical contact, and 68 (64.8%) reported no changes in non-sexual physical contact.

In the early phase of the pandemic (January-March), half of the PrEP users with reduced non-sexual contacts, also reported a decreased number of sexual partners. Consistently, during the lockdown (mid-March to May), 76.5% of the PrEP users with reduced non-sexual contacts also decreased their sexual partners. Similar trends could also be observed in PrEP users with no reduction in non-sexual contacts. 47 (69.1%) reduced the number of sexual partners during the early phase of the pandemic, and 55 (80.9%) during lockdown. Taken together, PrEP users with and without reduction in their non-sexual contacts both reported a reduction in their sexual contacts from January to March 2020 *(p* = *0.147*) and during the months of lockdown *(p* = *0.592)*.

All the participants who reported an increase in non-sexual contacts [3 (2.9%)], also reduced the number of sexual partners from early pandemic on.

### Frequency of STIs among the PrEP users

Quarterly PrEP appointments at WIR included STI-testing for *Neisseria gonorrhoeae* (*NG), Chlamydia trachomatis* (*CT), Mycoplasma genitalium* (*MG)* and *Treponema pallidum (TP)*. During the first lockdown in 2020, 27 participants were consulted *via* telephone, and received their PrEP prescription by mail. Accordingly, they were not tested for STIs until the next scheduled appointment until after the re-opening of WIR. Among them, 10 were diagnosed with an STI at their next appointment. Throughout the four quarters of 2020, more than every fourth PrEP-using MSM had an STI (Q1: 32.1%, Q2: 25.3%, Q3: 26.3%, Q4: 28.4%). In the pre-pandemic period, throughout the four quarters of 2018, 25.0–36.9% of the PrEP-using MSM had an STI. In essence, there was no significant difference in the proportion of STI positive PrEP-using MSM between 2018 and 2020 *(p* = *0.817)*.

During the pandemic year (2020), the most frequent STI was MG (38.5% of all positive STI tests), followed by CT (29.9%), NG (21.3%) and TP (11.0%).

Further, PrEP using MSM with reduced partner numbers demonstrated no difference in STI diagnosis during the first lockdown (mid-March to May), when compared to participants with the same or higher partner numbers *[X*^2^*(1)* = *1.24, p* = *0.27]*.

### Frequency of condom use among PrEP users

Analyses of self-reported frequency of condom use demonstrated no significant difference during the pandemic year 2020. Over a third of the PrEP using MSM (32.2–38.3%) never used a condom for anal intercourse, 23.7–33.0% of participants reported rare condom use. The trend in condomless sex among PrEP using MSM was similar during the pre-pandemic year 2018.

### Trends in substance use during sex

**Figure 4:**
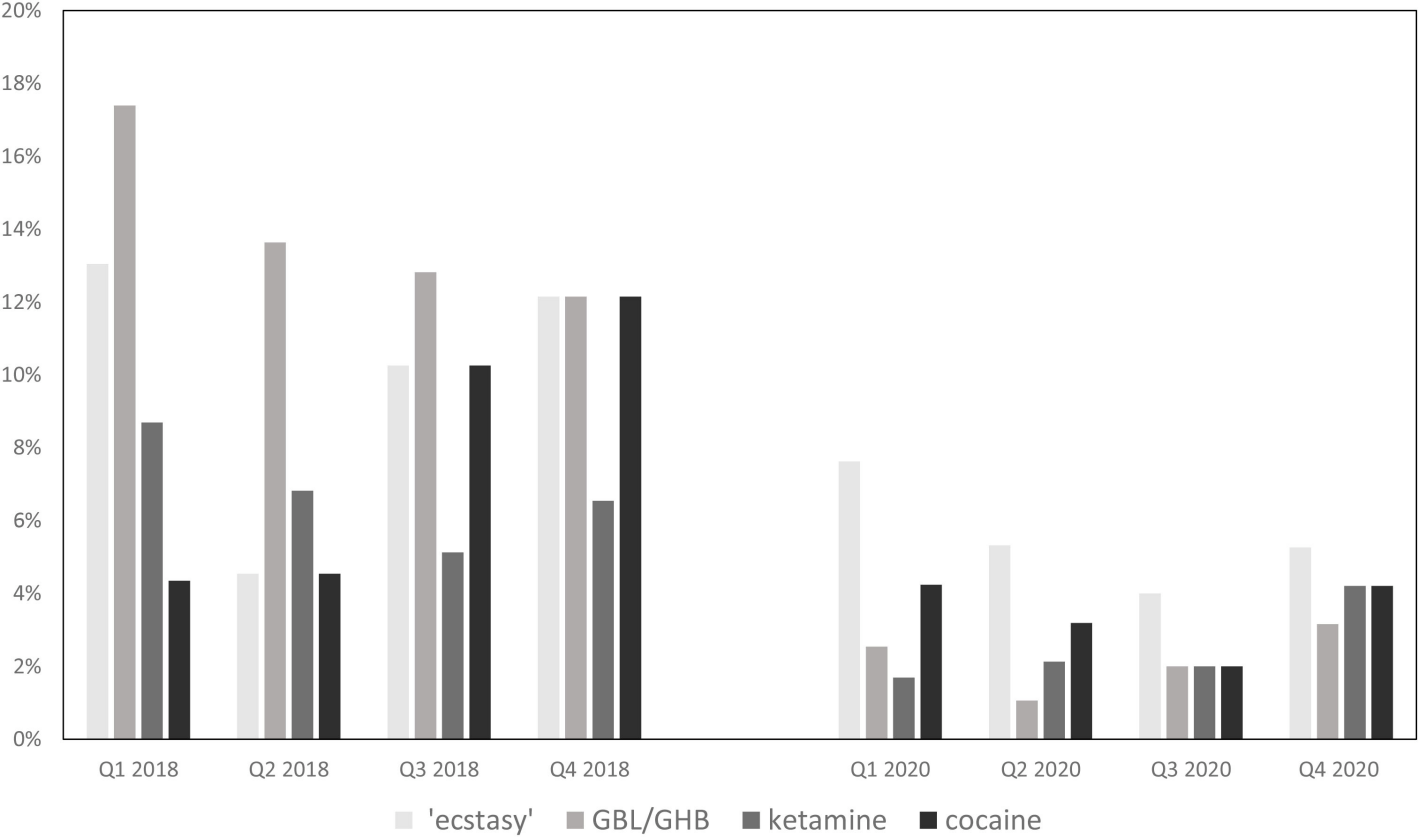
Sexualized drug use in the four quarters of 2018 vs. 2020.

Besides partner numbers and use of condoms during anal intercourse, substance use is another important component of sexual behavior. Figure 4 summarizes the proportions of PrEP using MSM who consumed psychoactive substances during sex in 2018 and 2020.

In 2020, across the four quarters, no significant differences in substance use were observed. Over the year, between 55.9%-67.4% of the PrEP users consumed substances during sex, among them alcohol, cannabis, poppers, potency increasing supplements and Chemsex drugs like “ecstasy,” GBL/GHB, ketamine, cocaine, and/or 3MMC. The most consumed drug was alcohol (32.0%−43.6%), followed by poppers (27.1–31.9%), cannabis (12.7–17.9%) and PDE5-inhibitors like sildenafil (11.9–17.9%). Numbers of PrEP users using Chemsex drugs were continuously low with no significant changes across the four quarters. Between 4 and 9 (4.0–7.6%) users stated to have taken “ecstasy” in 2020. Less than 4 users (0.8–4.2%) consumed one of the following Chemsex drugs: GBL/GHB, ketamine, cocaine, and/or 3MMC.

In comparison, PrEP users in 2018 reported higher levels of substance use. Over the year, three out of four participants (72.7–73.9%) reported having consumed substances of any kind during sex. In particular, alcohol (45.5–51.4%), poppers (47.8–60.3%), PDE5-inhibitors (27.3–35.9%) and cannabis (13.6%-21.7%) were used more commonly. Similar results were observed for Chemsex drugs: Up to 13 users (12.1%) stated use of “ecstasy,” cocaine and GBL/GHB and up to 7 (6.5%) used ketamine during sex in 2018. While in 2020 the maximum number of PrEP users who engaged in Chemsex was 13 (13.7%), up to 25 (23.4%) of PrEP users reported Chemsex in 2018.

Taken together, PrEP using MSM engaged in lower substance use during the pandemic year 2020 compared to the pre-pandemic year 2018.

### SARS-CoV-2 antibodies

From November 2020 to March 2021, a test for SARS-CoV-2 antibodies was conducted from the blood samples of PrEP using MSM. None of them reported having had a SARS-CoV-2 infection, nevertheless 3 (3.15%) participants were SARS-CoV-2 antibody positive. A few reported having experienced COVID-19-like symptoms but were negative for SARS-CoV-2 antibodies. Among the 3 SARS-CoV-2 antibody positive participants, 2 of them used daily PrEP during the pandemic, while the other participant reported periods of intermittent PrEP intake. Although tentatively promising, the data is insufficient to derive any conclusive association between oral use of PrEP and decreased infectability to COVID-19.

## Discussion

Our study attempted to understand the impact of the COVID-19 pandemic on HIV PrEP uptake among the MSM population, changes in their sexual behaviors, and HIV/STI testing profile. As yet consequences of health care disruptions during lockdown remain unexplored in Germany, this study warrants a precise contextualization of utilization of PrEP services, sexual behavior of PrEP-using MSM and HIV/STI testing services during the pandemic relative to the pre-pandemic period. Significant differences could be found regarding the intake regimen as well as a decrease in the number of sexual partners. However, the proportion of STI was rather stable. Germany reported a total of 3,111 new HIV cases for the year 2019 whereas only a total of 2,454 was reported for 2020, corresponding to a 21% decrease in new HIV cases from 2019 to 2020 (1). It is likely that the COVID-19 pandemic associated contact reduction has contributed to such a decline in new HIV diagnoses in 2020.

Sociodemographic data of our study cohort reveal that PrEP users in Germany are mostly well educated and financially independent individuals. A quarter of our PrEP users were still in their formative years as in schools, universities or in vocational training, thus representative of a relatively young cross-section of the population. The median age of our study participants were in mid-thirties, as was the case in similar studies exploring the pandemic's impact on PrEP users from clinics in Melbourne (12), Wales (13) or in exclusively online studies from Southern United States (14) wherein the median participant age was 26.

PrEP remained at continuous demand at WIR during the COVID-19 pandemic: A third of PrEP users reported daily use of PrEP both during the 1st lockdown (2^nd^ quarter 2020) and during the rather “light” lockdown (4^th^ quarter 2020), while more than half of them reported daily PrEP during summer 2020 (3^rd^ quarter). As opposed to previous years, in 2020 (including the months of lockdown and closure of the health center) first-time PrEP initiations were highly demanded. Similar need for PrEP supply during the pandemic has also been observed in other studies (12, 14, 15). However, the proportion of PrEP users reporting daily PrEP during 2020 varied across these studies from 58.8% in Australia (15) to 91% in the Southern United States (14). National differences in pandemic containment and contact restrictions might have contributed to these differences.

In our cohort of PrEP-using MSM, we found that the COVID-19 pandemic has impacted patterns of PrEP use: A third of participants paused PrEP during the pandemic as opposed to a proportion of 18% in 2018 (due to reasons unrelated to the pandemic), and a higher rate of transitioning to an intermittent PrEP intake regimen than in 2018 was also observed at our center. Reduced opportunities for casual sex during the pandemic might have been the major contributing factor for reducing PrEP use. These trends emphasize the increased need for adjustable PrEP medication (beyond license) with flexible regimens after an informed and realistic self-assessment of sexual behavior.

The COVID-19 pandemic has impacted sexual behavior among PrEP-using MSM in Germany. During the pandemic year 2020, most of the study participants reported lower numbers of sexual partners in comparison to pre-pandemic year (2018). This was regardless of a reduction in their non-sexual contacts. That said, we could not determine any correlation between social distancing and reduction in sexual contacts. These observations extend the body of literature which has also documented reductions in sexual risk behavior among MSM during the pandemic, however, such reductions may be short-lived (12, 13, 16). In contrast to a study from Amsterdam (17) showing a decrease in condom use during 2020, the frequency of reported condom use in study cohort showed no significant changes during the pandemic. Further, despite a smaller number of PrEP users with more than 20 partners during the current pandemic in Germany, our data does not indicate any corresponding variation in the amount of substance use during sexual activity. In contrast, Stephenson et al. reported an increase of drug intake during the pandemic in the US that was associable with an increase in casual sex partners (16).

To contextualize these findings relating to utilization of PrEP services and sexual behavior, we also examined changes in proportion of positive STI tests. Although a decrease in partner numbers was detectable in our findings, positive STI tests during the pandemic period (2020) remained similar to the pre-pandemic period (2018). Further, PrEP users with reduced partner numbers did not demonstrate less STI diagnoses, emphasizing the need for continued sexual health monitoring during pandemic scenarios. That said, a network-based model of co-circulating HIV, gonorrhea, and chlamydia among 103,000 MSM in the US projected that if sexual risk behavior rebounds while service interruption persists, an excess of hundreds of HIV cases and thousands of STI cases is inevitable over the next 5 years (18). Therefore, a systematic STI counseling in combination with PrEP initiations and reducing PrEP service interruptions seem essential to minimize indirect effect of the COVID-19 pandemic on the HIV/STI epidemics. MG as the most frequent STI among PrEP using MSM in the pandemic year 2020 is consistent with a nation-wide study in Germany (19), demonstrating high prevalence of STI, especially MG, among PrEP users. High frequency of MG as observed during the pandemic period may be due to high proportion of asymptomatic infections among PrEP users, and therefore delayed diagnosis and treatment. Hence, regular screenings must proceed also in pandemic scenarios.

Our findings taken together with similar studies from Australia (12), Wales (13), USA (16), seem to reveal diverse reactions to the pandemic when it comes to sexual behavior and STI testing within PrEP-taking populations, suggesting that living environments, different health policies, cultural influences and supply with prevention services can change how an individual's sexual health is affected by the pandemic.

As HIV prevention services like PrEP are not the first priority in an university hospital, individual personal capacities consumed by other challenges during the pandemic may also have resulted in decreasing PrEP access and HIV/STI testing (14, 20–22). Consistent with this, we observe that more than a quarter of the participants in our study did not present for follow-up after the first lockdown. Despite these challenges more than a third of them could be encouraged to schedule a new appointment for HIV/STI-testing and PrEP prescription, emphasizing the feasibility of a recall system to increase retention and adherence of the clientele. As this is an unprecedented global situation, telemedicine has an imperative role in allowing continued health support at times of lockdown and preventing worsening of the sexual, mental, and physical health during the pandemic. Tele-health services as conducted in our health center during the months of lockdown appear to be an effective tool for providing PrEP care in times of social distancing. For longer lasting periods of contact reduction, PrEP prescriptions need to be regularly scheduled with home-based self-sampled STI tests and personal assessment of sexual risk behavior to prevent delay in STI diagnosis. Although home-based self-sampling kits and a digital test for personal sexual risk behavior are already voluntarily available at the WIR center, implementation of such a strategy is not without its own challenges. In the US, a successful pilot study about use of home-based self-sampling revealed that: (1) clear instructions i.e., with a video demonstration are needed to facilitate self testing and (2) in case of a reactive test, a responsive protocol for immediate contact to health care services for information and treatment should be provided (23). If logistical and infrastructural solutions to these challenges can be put in place, a remote offer of digital PrEP prescription coupled to home-based tests could also be contemplated in Germany. For those MSM who were not primarily reachable through the offer of digital PrEP prescription, our recall system demonstrated promising results toward increasing PrEP prescription and adherence as well as utilization of other preventive care. Therefore, consistent with other programs (16, 24) “telePrEP” might be a promising facility for low-threshold access to PrEP. Nevertheless, for both telePrEP and remote offer of digital PrEP post home-based self-sampling, renal function testing remains another practical challenge.

Young, socially and sexually active PrEP users as in our study cohort can be assumed to have a larger number of social contacts, and during the current pandemic this exposes them to an increased risk of contracting SARS-CoV-2 infection. Two components of PrEP, tenofovir disoproxil fumarate (TDF) and emtricitabine (FTC), have been earmarked as potential therapeutics against SARS-CoV-2 (25), but attempts to correlate oral use of PrEP and clinical manifestations of COVID-19 has yielded conflicting conclusions (26–28). The positive SARS-CoV-2 serostatus of two participants with continuous PrEP intake regimen in our study cohort suggest that TDF and FTC may not be an efficient prophylaxis for SARS-CoV-2 infection. Nevertheless, since these participants did not report any symptoms, an attenuating effect on the course of disease can be hypothesized and hence provides an interesting area for future investigations.

Although our study is a premier study exploring the impact of COVID-19 pandemic on a PrEP-taking population in Germany, there are several limitations to it. Data was obtained from a monocentric study in one particular region of Germany and hence may not be generalizable to all PrEP users. Data of sexual behavior were self-reported by participants and may be subject to social desirability bias. During the pandemic, contact restriction measures often changed weekly. A web-based survey undertaken more frequently than every 3 months might have given more detailed information about impact of COVID measures on PrEP usage. Participants were predominantly financially secure and highly educated. A more socio-economically diverse sample might have shown greater variation in the consequences of COVID-19 and uncover structural vulnerabilities.

## Data availability statement

The raw data supporting the conclusions of this article will be made available by the authors, without undue reservation.

## Ethics statement

The studies involving human participants were reviewed and approved by Ethics Committee of the Medical Faculty at Ruhr-Universität Bochum (Request-number 17-6208-BR). The patients/participants provided their written informed consent to participate in this study.

## Author contributions

AP, AS-R, NB, and MU conceived the project and designed the study. AP, IM, PA, and MU were involved in the data collection. AK and JW helped with recruitment of participants. CT and DM provided the possibility to conduct the laboratory tests. MU conducted the statistical analyses, the analytical methods were verified by KS. The manuscript is written by MU in consultation with AP, AS-R, SN, AS, and NB. All authors discussed the results and provided critical feedback.

## Funding

We acknowledge support by the Open Access Publication Funds of the Ruhr-Universität Bochum. The study was supported by FoRUM of the Ruhr-Universität Bochum (Grant Number: F999N-20). This work was presented, in part, at the 10th German-Austrian Aids-Congress from the 25th-27th of March 2021 in form of a poster.

## Conflict of interest

The authors declare that the research was conducted in the absence of any commercial or financial relationships that could be construed as a potential conflict of interest.

## Publisher's note

All claims expressed in this article are solely those of the authors and do not necessarily represent those of their affiliated organizations, or those of the publisher, the editors and the reviewers. Any product that may be evaluated in this article, or claim that may be made by its manufacturer, is not guaranteed or endorsed by the publisher.
